# Appendix to the Second Edition of a Series of Observations on Strictures, &c.

**Published:** 1830-09

**Authors:** 


### BIBLIOGRAPHICAL NOTICES.

### *Appendix to the Second Edition of a Series of Observations on Strictures, &c.* By R. A. STAFFORD.

THE continued success Mr. STAFFORD has met with in the treatment of strictures by the lancetted stilettes, now enables him to add eighteen cases to those he before published. Mr. Stafford has now operated on upwards of forty cases of permanent stricture, "of the worst description, without a single failure." He assures us that in no instance has there been a false passage made, nor has the cutting through the contracted part either caused pain, hemorrhage, inflammation, or any other unfavorable symptom. The hardened structure which composed the stricture has always been absorbed, and he has never yet heard of a return of the complaint after this treatment. In addition to the unvaried success in the use of these instruments in stricture, Mr. S. has, on two different occasions, divided through an enlarged third lobe of the prostate gland, which on the one hand caused total, and in the other partial, retention of urine. In both these cases the disease subsided, and the patients recovered the complete power of the bladder.

Mr. Stafford states that there is but very trifling inconvenience caused by the use of the instruments. He has not always found it necessary, as he formerly recommended, to confine the patients, to apply leeches, or to leave the catheter after the operation in the urethra. "They have, on the following day, usually gone about their occupations, and it has only been necessary to pass a bougie daily for a short time, and afterwards three times a week, until the cure has been completed."

We must refer to our Journal for September 1828, and June 1829, for an account of the former editions of Mr. Stafford's work on the subject of Strictures of the Urethra. As in the latter Number several cases are detailed which were successfully treated by the lancetted stilette, we do not think it necessary to give any of the cases from the present Appendix.

We must once more be allowed to urge the necessity for the utmost caution in the use of these instruments.

---

Mr. Wardrop will be much gratified to hear that a brief essay will shortly be published on the subject of Cataract. The means of detecting the kind of cataract, or disorganization of the eye, *before* an operation, will be particularly pointed out. A case will be related, the details of which are very curious, and which cannot fail to be interesting to oculists in general. It may be a satisfaction to Mr. Wardrop to be informed that the same practitioner, the *presumed* "party writer," still continues in attendance upon this patient, and that she has been seen by some experienced oculists, who are of opinion that something may yet be done to relieve her. When the case is completed, it will be immediately given to the profession, together with a few comments which will convey some *moral* as well as medical instruction.

---

In the press, the Natural History of Poisons, vol. i. with a Plate. Contents: Physiology of Life, Secret and Slow Poisons, Epidemic and Endemic Diseases, Contagion, American and Arrow Poisons, Serpent Poisons, Pleasures and Pains of Opium. By John Murray, Esq. F.L.S. &c.

